# Generalised joint hypermobility and shoulder joint hypermobility, – risk of upper body musculoskeletal symptoms and reduced quality of life in the general population

**DOI:** 10.1186/s12891-017-1595-0

**Published:** 2017-05-30

**Authors:** Birgit Juul-Kristensen, Lasse Østengaard, Sebrina Hansen, Eleanor Boyle, Tina Junge, Lise Hestbaek

**Affiliations:** 1 Institute of Sports Science and Clinical Biomechanics, University of Southern Denmark, Odense, Denmark; 2 Dalla Lana School of Public Health, University of Toronto, Toronto, Canada; 3 Health Sciences Research Centre, University College Lillebaelt, Odense, Denmark

**Keywords:** Generalised joint hypermobility, Shoulder joint hypermobility, Upper body musculoskeletal symptoms, shoulder symptoms, Neck symptoms, Epidemiology, Quality of life

## Abstract

**Background:**

Generalised Joint Hypermobility (GJH) is a hereditary condition with an ability to exceed the joints beyond the normal range. The prevalence of GJH in the adult population and its impact on upper body musculoskeletal health and quality of life has mostly been studied in selected populations. The aims of this study were therefore, firstly to study the prevalence of GJH and GJH including shoulder hypermobility (GJHS), in the general Danish adult population; secondly to test the associations between GJH or GJHS and upper body musculoskeletal symptoms and health-related quality of life (HRQoL).

**Methods:**

The study was cross-sectional where 2072 participants, aged 25–65, randomly extracted from the Danish Civil Registration System), were invited to answer a questionnaire battery (Five-Part Questionnaire for classification of GJH, Standardised Nordic Questionnaire for musculoskeletal symptoms, EuroQoL-5D for HRQoL).

**Results:**

Totally 1006 (49%) participants responded. The prevalence of GJH and GJHS were 30% (*n* = 300) and 5% (*n* = 51), respectively. Compared with Non GJH (NGJH), participants with GJH and GJHS had Odds Ratio (OR) of 1.5-3.5 for upper body musculoskeletal symptoms within the last 12 months (mostly shoulders and hands/wrists). GJH and GJHS also had OR 1.6–4.4 for being prevented from usual activities, mostly due to shoulder and neck symptoms. Furthermore, GJH and GJHS had OR 2.2–3.1 for upper body musculoskeletal symptoms lasting for more than 90 days (neck, shoulders, hand/wrists), and 1.5–3.5 for reduced HRQoL (all dimensions, but anxiety/depression) compared with NGJH. Generally, most OR for GJHS were about twice as high as for those having GJH alone.

**Conclusions:**

GJH and GJHS are frequently self-reported musculoskeletal conditions in the Danish adult population. Compared with NGJH, GJH and especially GJHS, present with higher OR for upper body musculoskeletal symptoms, more severe symptoms and decreased HRQoL.

## Background

Musculoskeletal symptoms like pain, ache and discomfort is a burden for society due to its enormous societal costs. One of the musculoskeletal conditions, Generalised Joint Hypermobility (GJH), is a hereditary condition with an exaggerated ability to exceed the joints beyond the normal range of motion, defined by a certain number of positive joint mobility tests [1]. GJH has been found to be associated with musculoskeletal pain [2, 3], fibromyalgia [4], anxiety [5, 6], and musculoskeletal injuries [7]. GJH is even anticipated to be a risk factor for premature osteoarthritis [2], but this has never been confirmed.

Prevalence rates of adults with GJH measured clinically vary from 2% to 57% and these are dependent upon the test and criteria used, and the population investigated [2, 8]. High prevalence rates of GJH have been identified within specific sports/performing art activities, such as ballet dancing, gymnastics, swimming, and among musicians [9–12]. Most studies reporting prevalence of GJH include study samples from specific ethnic areas, age groups, or participants from specific health care clinics. But prevalence rates of GJH in a general adult population are lacking.

Tests for classifying GJH by clinical tests are the Beighton score, consisting of nine tests of satisfactory reproducibility [1, 13, 14]. For use in epidemiological studies, a validated questionnaire, the Five-part Questionnaire (5-PQ), has been developed taking actual, as well as historical information of hypermobility into consideration [3, 15, 16]. Using the 5-PQ the British population prevalence of GJH was 18% (based on physician consultations of 46,000 adults already included in a study of chronic widespread pain) [17], and 37% (based on 2,523 Brazilian university students) [15]. Due to the current prevalence rates being based only on selected populations, there is a need for a solid estimate of the prevalence in a random sample of the general adult population.

Previous studies have found an association between GJH and self-reported shoulder injuries in adult patient populations (e.g. Joint Hypermobility Syndrome and Ehlers Danlos Syndrome, Hypermobile Type) [18, 19], patients from emergency units with GJH/GJH with shoulder joint hypermobility (GJHS) and traumatic shoulder injuries [20], and in non-patient populations like military personnel [21], and adolescents with GJH [22]. Again, these associations were primarily studied in selected populations. In the general working population one of the contributors to neck/shoulder symptoms is reported to be work-related exposure [23, 24], but GJH/GJHS as another potential contributor to neck/shoulder symptoms in the general population is yet to be explored.

In summary, since former studies have reported prevalence of GJH, and an association between GJH and musculoskeletal symptoms using selected study-samples well known in the health care, there is potential risk of over-reporting. Therefore, the aim of this study was to determine the prevalence of GJH and GJHS in the general adult population in Denmark, and further investigate the association of GJH/GJHS with musculoskeletal symptoms in the upper part of the body, as well as with HRQoL.

## Methods

### Population

The study was cross-sectional and *the target population* was adults aged 25 to 65 years, living with a permanent home address in Denmark on January 2015. The data collection period was from January 2015 to June 2015.

For the *sampling population* an external institution, the governmental health authority State Serum Institute, approved the project (accept number FSEID-00001211, Date 24-11-2014), and accepted to extract a random list of names and home addresses of the adult population included in the Danish Civil Registration System (DCRS), a register of all residents in Denmark.

The *sampling method* included sending out a posted invitation letter about the study to everyone on the random list. The letter contained an internet link with a personal code to an online questionnaire that was hosted by SurveyXact [25] and instructions on how to fill out the questionnaire. Using a modified Dillman approach to increase response rates [26], non-responders received email reminders three and six weeks after the first invitation letter. The Regional Scientific Ethical Committee for Southern Denmark did not consider this study to be invasive and therefore, no ethics approval was warranted (12/5-2014; komite@rsyd.dk). Informed consent to participate according to the Declaration of Helsinki [27] was assumed based on a returned completed questionnaire.

### Questionnaire battery

The questionnaire battery contained the following questionnaires and questions: 5-PQ, Standardised Nordic Questionnaire (SNQ), EuroQoL-5D-5 L (EQ-5D-5 L), demographics (e.g., age, sex and current work status with response options of ‘employed, student, unemployed, absent due to illness, retired, early retired, or other’) and anthropometric questions (e.g., height and weight).

The 5-PQ consists of five questions that are used to determine the presence of GJH [3]. The questions are: Q1)’Can you now (or could you ever) place your hands flat on the floor without bending your knees?’ (Yes/No), Q2) ‘Can you now (or could you ever) bend your thumb to touch your forearm?’ (Yes/No), Q3) ‘As a child did you amuse your friends by contorting your body into strange shapes or could you do the splits?’ (Yes/No), Q4) ‘As a child or teenager did your shoulder or kneecap dislocate on more than one occasion?’ (Yes/No), and Q5) ‘Do you consider yourself double-jointed?’ (Yes/No). The 5-PQ has shown satisfactory test-retest reliability and criterion validity compared with clinical tests for GJH [15, 16]. Further, the 5-PQ has been linguistically translated and cross-culturally tested in a Danish patient and non-patient adult population [28]. The cut-point of two positive answers out of the five questions has been defined as the criteria for GJH. The 5-PQ questionnaire has been validated in two cohorts composed of patients and non-patients with sensitivities of 77% and 85% and specificities of 80% and 89% when compared against the gold standard of a clinical examination [3].

To determine the prevalence of GJHS the following question about shoulder joint hypermobility was included in the questionnaire battery: ‘Do you have increased range of motion, or are you loose in one or both shoulders?’ (Yes/No). This question has not undergone psychometric testing, but is based on current clinical knowledge and two previous studies that showed significant associations between GJH/GJHS and shoulder dislocations/subluxations [20, 21]. Participants who answered ‘*Yes*’ to the previous mentioned question, were classified as GJHS, provided that they were also categorized as having GJH.

The SNQ is a widely used questionnaire for assessing presence and severity of musculoskeletal trouble, which we refer to as symptoms in this paper [29]. To illustrate the body region that each question pertains to a body diagram with the appropriate area(s) (i.e., neck, shoulders, elbows, wrists/hands) shaded in is located next to each question. SNQ has obtained satisfactory reliability and validity against clinical examinations [30–32]. Participants were asked whether they had any musculoskeletal symptoms (pain, ache, discomfort) in these body regions within the last year (yes/no) and about the severity. The questions about severity included whether their symptoms had prevented them from performing their usual activities (at home/outside their home) (yes/no), and for how many days they had had these symptoms within the last year (0 days/1–7 days/8–30 days/31–90 days/above 90 days). For the analysis purpose, the number of days was collapsed into: 0 days, 1 to 90 days and more than 90 days, the latter being indicative of a chronic musculoskeletal condition [33, 34].

The EQ-5D-5 L consists of two parts. The first part is composed of five dimensions (i.e., mobility, self-care, usual activities, pain/discomfort and anxiety/depression) about today’s perceived health status [35, 36]. Each dimension is rated using a five-level ordinal scale as follows: 1) no problems, 2) slight problems, 3) moderate problems, 4) severe problems, and 5) extreme problems [37]. The Index calculator of the EuroQol Research Foundation was used to calculate the total index-score of the HRQoL, ranging from one to below null [38]. A score of one indicates that the participants perceived their health at the best possible state and a score below null that the participants perceived their health worse than death, with null as the reference score of death. In patients with shoulder instability problems, EQ-5D-5 L has shown satisfactory psychometric properties [39, 40].

The second part of the EQ-5D-5 L involves the participants marking their today’s perceived health on a visual analogue scale (VAS), ranging from ‘the best health you can imagine’ to ‘the worst health you can imagine’, called the EuroQol VAS. This scale ranges from 0 to 100, with a score of 100 as the best possible health and null as the worst possible health.

### Statistical analyses

Complete case analysis was conducted because 17 participants did not complete the entire questionnaire. We decided not to do imputation because we felt the missing was not random and we did not want to introduce any bias to the data [41].

Data was tested for normality (Shapiro Wilk, histogram distributions) and was found not to be normal. Descriptive statistics displaying median with interquartile range (IQR) for continuous data and percentage with 95% confidence interval for categorical data were used to present demographic data. Group differences on demographics in participants with Non Generalised Joint Hypermobility (NGJH) versus GJH, and NGJH versus GJHS were tested with Wilcoxon rank-sum (Mann–Whitney *U*-test) for continuous data and with Chi-square or Fisher’s exact test for categorical data. Due to the number of comparisons (68 comparisons: 2 groups (GJH and GJHS), 2 models (crude and adjusted models), 4 body regions/5 dimensions in EQ-5D-5 L, four questions (prevalence within the last year, being prevented from performing their usual activities, having had musculoskeletal symptoms for more than 90 days, EQ-5D-5 L)), the level of significance for all analyses was adjusted by the Bonferroni method, implying that the statistical level of significance corresponds to p < 0.025. To limit the number of tables, data on prevalence and OR for musculoskeletal symptoms lasting for more than 90 days per year have only been presented in the text.

Tests for associations between GJH/GJHS and the nominal/ordinal outcomes for reporting musculoskeletal symptoms, in addition to the five dimensions of the EQ-5D-5 L, were performed with either logistic or ordered/multinomial logistic regression analyses (crude and adjusted models where sex was controlled), displayed as OR and 95% confidence Interval (95% CI).

Sample size calculation was based on an estimated prevalence of musculoskeletal symptoms in the general adult population in Denmark, corresponding to approximately 15% [42], and an estimated prevalence of musculoskeletal symptoms in adults with GJH of 30% [19]. The prevalence of GJH was set to a ratio of 1:6, which equals 16.6%. A two-sample proportion Chi-square test, with significance level of 5% and power of 80% in a two-sided test, showed that 518 respondents were required to detect a significant difference in musculoskeletal symptoms between those with GJH/GJHS and NGJH. Due to an expected response rate of 28% [17], the current survey was sent out to four times as many participants as required (4 × 518), corresponding to 2072 participants. All statistical analyses were conducted in STATA 13, StataCorp. 2013.

## Results

**Fig. 1:**
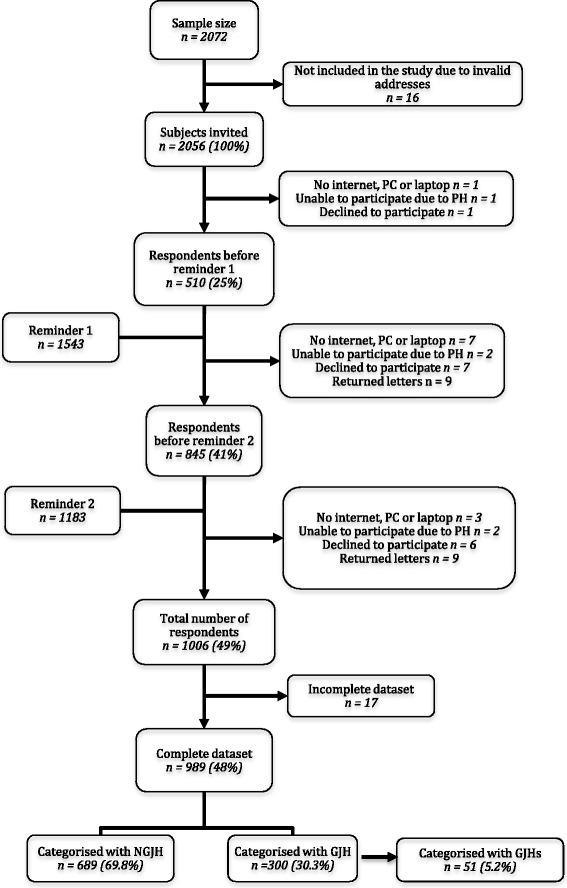
Flowchart of the participant recruitment

Of the 2072 randomly generated names and addresses from the DCRS, 16 addresses were found to be invalid. Therefore, a total of 2056 participants were invited to participate and 1006 participants responded, resulting in a response rate of 49%, of which 989 (98%) participants filled out the entire questionnaire. Fourteen (1.4%) participants declined to participate, 16 (1.6%) were unable to participate, 18 letters were returned to sender (1.8%), and 1050 (51%) did not access the questionnaire using the Internet address (Fig. 1).

The median age of the cohort was 50 years (IQR 40–57 years) and participants with GJH were slightly younger (48 years vs 50 years; *p* = 0.025) and more often females (GJH/GJHS 77% or 76% vs 47% in NGJH; *p* < 0.001) than participants with NGJH (Table 1). There were no significant group differences in Body Mass Index and work-related status.

**Table 1:** Demographics of participants with NGJH (Non Generalised Joint Hypermobility), GJH (Generalised Joint Hypermobility) and GJHS (GJH including shoulder joint hypermobility)

Variable	All (*n* = 989)	NGJH (*n* = 689)	GJH (*n* = 300)	*p*-value	GJHS (*n* = 51)	*p*-value
Women, % (no.)	55.9 (553)	47.3 (326)	75.7 (227)	<0.001*	76.5 (39)	<0.001*
Age, median (IQR)	50 (40–57)	50 (41–57)	48 (38–55.5)	0.025	46 (34–57)	0.145
BMI (Kg/m^2^), median (IQR)	25.4 (22.9–28.1)	25.5 (23.1–28.1)	25.1 (22.5–28.1)	0.151	24.5 (21.2–29.0)	0.284
Work-related status, % (no.)				0.188		0.149
Employed	71.2 (704)	72.3 (498)	68.7 (206)		54.9 (28)	
Un-employed	3.74 (37)	3.34 (23)	4.67 (14)		3.92 (2)	
Student	3.74 (37)	3.19 (22)	5.00 (15)		5.88 (3)	
Absent because of illness	1.82 (18)	1.60 (11)	2.33 (7)		3.92 (2)	
Early retired	3.94 (39)	3.19 (22)	5.67 (17)		7.84 (4)	
Retired	4.15 (41)	4.06 (28)	4.33 (13)		7.84 (4)	
Other	11.43 (113)	12.3 (85)	9.33 (28)		15.7 (8)	
EuroQol VAS, median (IQR)	85 (75–90)	85 (75–90)	80 (70–90)	<0.001*	75 (60–90)	<0.001*
EQ-5D Index, median (IQR)	0.86 (0.77–1)	0.86 (0.78–1)	0.82 (0.74–0.86)	<0.001*	0.79 (0.67–0.86)	<0.001*

*Indicates a statistical significant difference (*p*-value <0.025) between participants with NGJH and GJH, and between NGJH and GJHS

### Prevalence of GJH and GJHS

The prevalence of GJH was 30% (*n* = 300) and the prevalence of GJHS was 5% (*n* = 51) (Fig. 1). Of those classified with GJH (*n* = 300), almost all of the participants (90.3%, *n* = 271) answered ‘Yes’ to Q1) ‘Can you now (or could you ever) place your hands flat on the floor without bending your knees?’. For those classified with GJHS (*n* = 51), both Q1 and Q5, ‘Do you consider yourself double-jointed?’ (Q5), were most frequently answered positively, corresponding to 82.4% (*n* = 42) and 84.3% (*n* = 43), respectively (not in Tables).

### Musculoskeletal symptoms in the upper body regions

Participants with GJH and GJHS were more likely to report musculoskeletal symptoms within the last 12 months for the upper body sites than NGJH. The highest proportion of region specific musculoskeletal symptoms was for participants with GJHS. This was 76% for the shoulder (95%CI: 64–89) (not in tables). Overall, participants with GJHS had higher OR of having symptoms with their upper body regions (especially shoulders, hands/wrists) than NGJH participants (Table 2). The adjusted analyses were similar to the crude analyses except for the neck, which did not reach significance in the adjusted analyses.

**Table 2:** Crude (Model 1) and adjusted (Model 2) logistic regressions, with odds ratio (OR) and 95% confidence interval (95% CI), for musculoskeletal symptoms within the last 12 months, and for being prevented from performing their usual activities (at home/outside their home), due to musculoskeletal symptoms in the different body regions (neck, shoulders, elbows, hand/wrists) presented for participants with GJH (Generalised Joint Hypermobility) and GJHS (GJH including shoulder joint hypermobility), with NGJH (Non Generalised Joint Hypermobility) as reference group

Musculoskeletal symptoms last 12 mths	GJH		GJHS	
	Model 1 OR (95% CI)	Model 2 OR (95% CI)	Model 1 OR (95% CI)	Model 2 OR (95% CI)
At individual sites				
Neck	1.66 (1.24–2.23)*	1.34 (0.99–1.82)	1.77 (0.94–3.33)	1.49 (0.78–2.84)
Shoulders	1.68 (1.28–2.21)*	1.45 (1.09–1.92)*	3.79 (1.95–7.37)*	3.37 (1.72–6.58)*
Elbows	1.33 (0.97–1.83)	1.29 (0.93–1.80)	2.14 (1.17–3.91)*	2.08 (1.13–3.83)*
Hands/wrists	2.49 (1.87–3.32)*	2.24 (1.67–3.01)*	3.96 (2.22–7.08)*	3.53 (1.96–6.35)*
Prevented from performing their usual activities				
Neck	2.09 (1.51–2.90)*	1.82 (1.30–2.56)*	4.94 (2.75–8.90)*	4.41 (2.43–8.00)*
Shoulders	1.77 (1.28–2.44)*	1.55 (1.11–2.18)*	3.75 (2.08–6.75)*	3.35 (1.84–6.09)*
Elbows	1.43 (0.88–2.31)	1.37 (0.83–2.26)	2.86 (1.32–6.23)*	2.83 (1.28–6.27)*
Hands/wrists	2.02 (1.39–2.94)*	1.82 (1.23–2.69)*	2.60 (1.30–5.18)*	2.36 (1.17–4.76)*

*Indicates a *p*-value <0.025

The severity of the region specific musculoskeletal symptoms was determined by whether it prevented the participants from performing their usual daily activities (in/outside their home) and by the duration of their symptoms. Participants with GJH and GJHS were more likely to have difficulties performing their usual activities due to symptoms in their upper body regions (especially neck, shoulders), except for the elbows in GJH (not in tables). Neck and shoulder regions also had the highest OR for positive associations with difficulties in performing their usual activities, and the OR for GJHS were generally about twice as high than GJH only (Table 2).

With respect to musculoskeletal symptoms lasting for more than 90 days per year, only neck symptoms were reported significantly more often for GJH (24%) and for GJHS (32%) than for NGJH (15%) (not presented in Tables). Positive associations for musculoskeletal symptoms lasting for more than 90 days per year were seen in all regions (except for the elbow), with OR between 2.18 and 3.11 (not presented in Tables).

### Health related quality of life (HRQoL)

Participants with GJH (median EuroQoL VAS = 80) or with GJHS (median = 75) reported significantly lower HRQoL score than the NGJH participants (median = 85, *p* < 0.001) (Table 1). Similarly, the EQ-5D-5 L Index was significantly lower for the participants with either GJH or GJHS. The same was observed for each of the five dimensions of the EQ-5D-5 L Index. OR ranged from 2.8 to 3.5 for GJHS, and from 1.5 to 1.9 for GJH when compared with NGJH, showing OR for GJHS generally being twice as high as for those with GJH alone (Table 3).

**Table 3:** Crude (Model 1) and adjusted (Model 2) logistic regressions, with odds ratio (OR) and 95% confidence interval (95% CI), for problems in Health Related Quality of Life in the EQ-5D-5 L dimensions, presented for participants with Non Generalised Joint Hypermobility (NGJH) compared with GJH, and for NGJH compared with GJHS (GJH including shoulder joint hypermobility)

	GJH		GJHS	
EQ-5D-5 L dimensions	Model 1 OR (95% CI)	Model 2 OR (95% CI)	Model 1 OR (95% CI)	Model 2 OR (95% CI)
Mobility	1.48 (1.07–2.04)*	1.47 (1.05–2.06)*	3.36 (1.87–6.04)*	3.39 (1.87–6.16)*
Self-care	1.80 (1.14–2.86)*	1.83 (1.13–2.95)*	3.33 (1.57–7.07)*	3.49 (1.61–7.54)*
Usual activities	1.81 (1.36–2.41)*	1.85 (1.38–2.49)*	3.22 (1.81–5.73)*	3.35 (1.87–6.02)*
Pain/discomfort	1.81 (1.35–2.42)*	1.69 (1.25–2.28)*	2.98 (1.47–6.05)*	2.81 (1.38–5.73)*
Anxiety/depression	1.42 (1.04–1.94)	1.38 (1.00–1.91)	1.86 (1.01–3.42)	1.81 (0.97–3.36)

* Indicates a *p*-value <0.025

## Discussion

The estimated prevalence was 30% for GJH and 5% for GJHS. Compared with NGJH, participants with GJH and GJHS had higher odds for upper body musculoskeletal symptoms within the last 12 months (mostly in the shoulders and hands/wrists), and higher odds of being prevented from performing their usual activities in/outside their home (mostly due to neck and shoulder symptoms). Furthermore, GJH and GJHS had increased OR of 2.2-3.1 for upper body musculoskeletal symptoms in all regions lasting for more than 90 days (except in elbows), and both groups had increased OR for reduced HRQoL (except for anxiety/depression). Most OR for GJHS were about twice as high as for those having GJH alone.

Compared with other countries, the current prevalence of 30% is similar to a Brazilian study of university students that reported a prevalence of 37% using the 5-PQ (and in the same study 34% using the Beighton score of at least 4/9) [15]. However, the present prevalence is higher than a British survey of adults reporting a prevalence of 18% also using the 5-PQ [17]. This may be due to the participants in the British study were older than in the current study (median of 55 years versus 50 years in the current study), and thus recall bias of previous hypermobility abilities may have been different in the two studies, in addition to the fact that people become less hypermobile with age [2]. An uneven sex ratio (in all 56% women) is also likely a contributing factor to the higher estimation of GJH in the current study, because the prevalence of GJH is higher in women than in men [2]. However, the current sex ratio was similar to both the British and the Brazilian studies [15, 17]. The prevalence of GJH is furthermore known to be higher in non-Caucasians [43]. The Danish proportion of immigrants and their descendants (non-Caucasians) did only account for 11.6% of the total population at the time of the study, making the current study population fairly homogeneous with respect to ethnicity (Statistics Denmark, 2015). Thus a higher rate of non-Caucasians seems not likely to be a reason for the higher prevalence of GJH in Denmark than in the UK.

The prevalence of GJHS was 5%, but to our knowledge there has not been another study that has examined the prevalence of shoulder joint hypermobility in combination with GJH. Since neither the most often used clinical tool for classification of GJH (the Beighton score), nor the 5-PQ include a specific test/question for shoulder joint hypermobility, the current addition of a question targeting specifically the shoulder provides information that was previously unknown.

Generally, participants with GJH were significantly more likely to report musculoskeletal symptoms in the upper part of the body than NGJH. This is similar to previous studies that reported significant associations with musculoskeletal symptoms in GJH compared with NGJH [2, 8, 17, 18]. The severity of musculoskeletal symptoms was obvious in the current study, illustrated by the high proportion of participants with GJH/GJHS being prevented from performing their usual activities, and the proportion reporting chronic musculoskeletal symptoms (more than 90 days per year). The most frequent body regions preventing participants with GJHS from performing their usual activities (in/outside home) and with more than 90 days of symptoms were the shoulder and neck regions, possibly due to the hypermobile shoulder condition. Although GJH is a well-known risk factor for joint dislocation and soft tissue injuries (e.g. muscle, tendon and ligament injuries) [2], the information of the high presence of musculoskeletal symptoms among participants with GJH in Denmark and especially GJHS is new. It was anticipated that GJHS would be associated with musculoskeletal symptoms, especially in the shoulder, and this was mostly confirmed in the current study. Not surprisingly, participants with GJHS had about three fold higher odds of musculoskeletal symptoms in the shoulder and neck than NGJH. Symptoms in all other upper body regions could be due to compensatory movements resulting in overload.

The low HRQoL scores in participants with GJH are in agreement with a previous study of patients diagnosed with Ehlers Danlos Syndrome, Hypermobile Type [19], but even lower HRQoL in participants with GJHS has not been previously reported. Both patients diagnosed with shoulder instability and more severe conditions of hypermobility (e.g. Joint Hypermobility Syndrome and Ehlers Danlos Syndrome-Hypermobile Type) have been found to be associated with severely reduced HRQoL compared with NGJH [18, 44], which seems to support the current reduced HRQoL, especially in those with GJHS. Noteworthy and surprisingly, the EQ-5D-5 L’s physical activity dimension had the highest OR for decreased HRQoL in participants with GJHS. This underlines the high physical impact GJH and GJHS has on participants’ life. The current study found no associations for GJH and GJHS with the mental-related dimension of anxiety/depression, which has previously been reported in patients with Ehlers Danlos Syndrome, Hypermobile Type [19]. However, a recent study did also find no significant association between patients with Joint Hypermobility Syndrome/Ehlers Danlos Syndrome, Hypermobile Type and mental health, thereby supporting the current results further [18]. One of the reasons for the conflicting results of the impact GJH has on mental health [19] may be due to different methods of measuring mental health (the current one dimension of the EQ-5D-5 L versus the previously used four dimensions in SF-36).

The current response rate of 49% was fairly high, given only two reminders, and that the participants had to initiate the response to the electronic questionnaire, and it was actually higher than in the mentioned British population-based study (28%) [17]. Since the study had a focus on joint hypermobility, participants with no signs or knowledge of hypermobility may have been less likely to answer the questionnaire, with a risk of overestimating the prevalence. Unfortunately, since the random sample only included names and home addresses, no drop out analyses could be performed on demographics. However, previous studies using the same method (no aggressive protocol, no incentives, no sponsor sender, the national survey) have shown no response rate bias with regards to low response rates and skewed demographics (age, sex, marital status, education) [45, 46].

Selection bias was anticipated not to have influenced the results in relation to musculoskeletal symptoms and HRQoL, since the 12-month prevalence of musculoskeletal symptoms and activity limitations for the participants were in accordance with previous studies [17–19, 22], as was the distribution of responses to the HRQoL [18, 39, 40, 47, 19]. With the relatively long recall periods for both GJH (5-PQ: ever/now) and musculoskeletal symptoms (SNQ: last 12 months) recall bias may be present, but this does not seem likely, since satisfactory psychometric properties (reliability and validity) have been reported for both 5-PQ and SNQ [3, 15, 30, 32].

Although previous studies have investigated HRQoL and musculoskeletal symptoms in participants with Joint Hypermobility Syndrome/Ehlers Danlos Syndrome, Hypermobile Type, no previous study has used the SNQ and EQ-5D-5 L in a population-based study of GJH and GJHS. This makes it difficult directly to compare the current data with other studies.

The strength of this study was the random selection of the study sample at the population level and the extraction of data through a governmental institution, which resulted in a representative sample of adults living in Denmark during the study period. Furthermore, with the exception of the additional question on GJHS, only validated questionnaires were used in the current population-based survey [3, 40, 48–50]. Since both unadjusted and adjusted models showed almost the same pattern of significance, the current findings and associations appear to be consistent.

## Conclusions

GJH and GJHS are frequently self-reported musculoskeletal conditions in the general adult population. Compared with NGJH, GJH and especially GJHS present with higher odds and severity of upper body musculoskeletal symptoms and decreased HRQoL. Also, there is a need to study the prevalence of GJH including lower extremity hypermobility and the association to lower body musculoskeletal symptoms. Further, since the current study is a cross-sectional study, longitudinal studies are recommended to describe the onset, fluctuations and persistence of chronic musculoskeletal symptoms and osteoarthritis in the general adult population, and how musculoskeletal symptoms are associated with GJH through the life course.

## Abbreviations

5-PQ: Five-part questionnaire
DRCS: Danish civil registration system
EQ-5D-5 L: EuroQol 5D 5 L
GJH: Generalised joint hypermobility
GJHS: Generalised joint hypermobility and shoulder hypermobility
HRQoL: Health-related quality of life
IQR: Interquartile range
NGJH: Non generalised joint hypermobility
OR: Odds ratio
Q: Question
SNQ: Standardised nordic questionnaire

## References

[1] Remvig L, Jensen DV, Ward RC (2007). Are diagnostic criteria for general joint hypermobility and benign joint hypermobility syndrome based on reproducible and valid tests? A review of the literature. J Rheumatol.

[2] Remvig L, Jensen DV, Ward RC (2007). Epidemiology of general joint hypermobility and basis for the proposed criteria for benign joint hypermobility syndrome: review of the literature. J Rheumatol.

[3] Hakim AJ, Grahame R (2003). A simple questionnaire to detect hypermobility: an adjunct to the assessment of patients with diffuse musculoskeletal pain. IntJ Clin Pract.

[4] Acasuso-Diaz M, Collantes-Estevez E (1998). Joint hypermobility in patients with fibromyalgia syndrome. Arthritis Care Res.

[5] Bulbena A, Gago J, Pailhez G, Sperry L, Fullana MA, Vilarroya O (2011). Joint hypermobility syndrome is a risk factor trait for anxiety disorders: a 15-year follow-up cohort study. Gen Hosp Psychiatry.

[6] Smith TO, Easton V, Bacon H, Jerman E, Armon K, Poland F (2014). The relationship between benign joint hypermobility syndrome and psychological distress: a systematic review and meta-analysis. Rheumatology (Oxford).

[7] Pacey V, Nicholson LL, Adams RD, Munn J, Munns CF (2010). Generalized joint hypermobility and risk of lower limb joint injury during sport: a systematic review with meta-analysis. Am J Sports Med.

[8] Hakim A, Grahame R (2003). Joint hypermobility. Best Pract Res Clin Rheumatol.

[9] McCormack M, Briggs J, Hakim A, Grahame R (2004). Joint laxity and the benign joint hypermobility syndrome in student and professional ballet dancers. J Rheumatol.

[10] Pink MM, Tibone JE (2000). The painful shoulder in the swimming athlete. Orthop Clin North Am.

[11] Brandfonbrener AG (2002). Joint laxity and arm pain in a large clinical sample of musicians. Med Probl Perform Art.

[12] Larsson LG, Baum J, Mudholkar GS, Srivastava DK (1993). Hypermobility: Prevalence and features in a swedish population. Br J Rheumatol.

[13] Juul-Kristensen B, Rogind H, Jensen DV, Remvig L (2007). Inter-examiner reproducibility of tests and criteria for generalized joint hypermobility and benign joint hypermobility syndrome. Rheumatology (Oxford).

[14] Beighton P, Solomon L, Soskolne CL (1973). Articular mobility in an African population. Ann Rheum Dis.

[15] Moraes DA, Baptista CA, Crippa JA, Louzada-Junior P (2011). Translation into Brazilian Portuguese and validation of the five-part questionnaire for identifying hypermobility. Rev Bras Reumatol.

[16] Bulbena A, Mallorqui-Bague N, Pailhez G, Rosado S, Gonzalez I, Blanch-Rubio J (2014). Self-reported screening questionnaire for the assessment of Joint Hypermobility Syndrome (SQ-CH), a collagen condition, in Spanish population. Eur J Psychiat.

[17] Mulvey MR, Macfarlane GJ, Beasley M, Symmons DP, Lovell K, Keeley P (2013). Modest association of joint hypermobility with disabling and limiting musculoskeletal pain: results from a large-scale general population-based survey. Arthritis Care Res (Hoboken).

[18] Johannessen EC, Reiten HS, Løvaas H, Maeland S, Juul-Kristensen B (2016). Shoulder function, pain and health related quality of life in adults with Joint Hypermobility Syndrome/Ehlers-Danlos Syndrome-Hypermobility Type. Disabil Rehabil (epub ahead of print).

[19] Rombaut L, Malfait F, Cools A, De PA, Calders P (2010). Musculoskeletal complaints, physical activity and health-related quality of life among patients with the Ehlers-Danlos syndrome hypermobility type. Disabil Rehabil.

[20] Chahal J, Leiter J, McKee MD, Whelan DB (2010). Generalized ligamentous laxity as a predisposing factor for primary traumatic anterior shoulder dislocation. J Shoulder Elbow Surg.

[21] Cameron KL, Duffey ML, DeBerardino TM, Stoneman PD, Jones CJ, Owens BD (2010). Association of generalized joint hypermobility with a history of glenohumeral joint instability. J Athl Train.

[22] Tobias JH, Deere K, Palmer S, Clark EM, Clinch J. Hypermobility is a risk factor for musculoskeletal pain in adolescence: Findings from a prospective cohort study. Arthritis Rheum 2013;65:1107–15. 10.1002/art.37836 23450628

[23] Aptel M, Aublet-Cuvelier A, Cnockaert JC (2002). Work-related musculoskeletal disorders of the upper limb. Joint Bone Spine.

[24] Mehlum IS, Kjuus H, Veiersted KB, Wergeland E (2006). Self-reported work-related health problems from the Oslo Health Study. Occup Med (Lond).

[25] Rambøll. SurveyXact. 2014.

[26] Dillman DA (2007). Mail and Internet surveys : the tailored design method.

[27] Vollmann J, Winau R (1996). Informed consent in human experimentation before the Nuremberg code. BMJ.

[28] Hakim AJ. Approval of the Danish version of Five-Part Questionnaire (by Hakim AJ, Grahame R. A simple questionnaire to detect hypermobility: an adjunct to the assessment of patients with diffuse musculoskeletal pain. 2003). Personal communication 2014. 12723715

[29] Kuorinka I, Jonsson B, Kilbom A, Vinterberg H, Biering-Sorensen F, Andersson G (1987). Standardised Nordic questionnaires for the analysis of musculoskeletal symptoms. Appl Ergon.

[30] Palmer KT (2008). Diagnosing soft tissue rheumatic disorders of the upper limb in epidemiological studies of vibration-exposed populations. Int Arch Occup Environ Health.

[31] Juul-Kristensen B, Kadefors R, Hansen K, Bystrîm P, Sandsjî L, Sjõgaard G (2006). Clinical signs and physical function in neck and upper extremities among elderly female computer users: the NEW study. Eur J Appl Physiol.

[32] Palmer K, Smith G, Kellingray S, Cooper C (1999). Repeatability and validity of an upper limb and neck discomfort questionnaire: the utility of the standardized Nordic questionnaire. Occup Med (Lond).

[33] Von Korff M, Jensen MP, Karoly P (2000). Assessing global pain severity by self-report in clinical and health services research. Spine (Phila Pa 1976).

[34] Mallen C, Peat G, Thomas E, Croft P (2005). Severely disabling chronic pain in young adults: prevalence from a population-based postal survey in North Staffordshire. BMC Musculoskelet Disord.

[35] EuroQol G (1990). EuroQol--a new facility for the measurement of health-related quality of life. Health Policy.

[36] Rabin R, de Charro F (2001). EQ-5D: a measure of health status from the EuroQol Group. Ann Med.

[37] Janssen MF, Pickard AS, Golicki C, Gudex C, Niewada M, Scalone L (2012). Measurement properties of the EQ-5D-5 L compared to the EQ-5D-3 L across eight patient groups: a multi-country study. Qual Life Res.

[38] van Hout B, Janssen MF, Feng YS, Kohlmann T, Busschbach J, Golicki D (2012). Interim scoring for the EQ-5D-5 L: mapping the EQ-5D-5 L to EQ-5D-3 L value sets. Value Health.

[39] Hinz A, Kohlmann T, Stobel-Richter Y, Zenger M, Brahler E (2014). The quality of life questionnaire EQ-5D-5 L: psychometric properties and normative values for the general German population. Qual Life Res.

[40] Skare O, Liavaag S, Reikeras O, Mowinckel P, Brox JI (2013). Evaluation of Oxford instability shoulder score, Western Ontario shoulder instability index and Euroqol in patients with SLAP (superior labral anterior posterior) lesions or recurrent anterior dislocations of the shoulder. BMC res notes.

[41] Kristman VL, Manno M, Cote P (2005). Methods to account for attrition in longitudinal data: do they work? A simulation study. Eur J Epidemiol.

[42] Christensen AI, Ekholm O, Davidsen M, Juel K (2012). Sundhed og sygelighed i Danmark 2010 & udviklingen siden 1987.

[43] Beighton P, Grahame R, Bird H. Hypermobility of Joints. 2012.

[44] Gartsman GM, Brinker MR, Khan M, Karahan M (1998). Self-assessment of general health status in patients with five common shoulder conditions. J Shoulder Elbow Surg.

[45] Davern M (2013). Nonresponse rates are a problematic indicator of nonresponse bias in survey research. Health Serv Res.

[46] Groves RM, Couper MP, Presser S, Singer E, R. T, G.P. A et al. Experiments in producing nonresponse bias. Public Opin Q 2006;70: 16.

[47] Clemens S, Begum N, Harper C, Whitty JA, Scuffham PA (2014). A comparison of EQ-5D-3 L population norms in Queensland, Australia, estimated using utility value sets from Australia, the UK and USA. Qual Life Res.

[48] Crawford JO (2007). The Nordic Musculoskeletal Questionnaire. Occup Med.

[49] Wannstrom I, Peterson U, Asberg M, Nygren A, Gustavsson JP (2009). Psychometric properties of scales in the General Nordic Questionnaire for Psychological and Social Factors at Work (QPS): confirmatory factor analysis and prediction of certified long-term sickness absence. Scand J Psychol.

[50] Jonathan H. Tobias, Kevin Deere, Shea Palmer, Emma M. Clark, Jacqui Clinch. Joint Hypermobility Is a Risk Factor for Musculoskeletal Pain During Adolescence: Findings of a Prospective Cohort Study. Arthritis & Rheumatism. 2013;65(4):1107–15. 10.1002/art.37836 23450628

